# First report of disseminated *Mycobacterium abscessus* in an immunocompetent adult patient in Oman

**DOI:** 10.1016/j.ijregi.2022.01.010

**Published:** 2022-01-20

**Authors:** Ahmed Al Mamari, Wafa Al Tamtami, Kawther Al A’amri, Hassan Al Lawati, Said Al Kalbani, Issam Al Muqbali

**Affiliations:** a Ministry of Health, Muscat, Oman; b Armed Forces Hospital, Muscat, Oman

**Keywords:** *Mycobacterium abscessus*, Disseminated Mycobacterium infection, Septic arthritis, Oman, Rapid-growing mycobacteria, Atypical mycobacteria, Non-tuberculous mycobacteria, Immunocompetent patient, RGM, rapid-growing mycobacterium, ROM, range of motion, CRP, C-reactive protein, NTM, non-tuberculous mycobacterium, ID, infectious diseases, MALDI-TOF MS, matrix-assisted laser desorption/ionization-time of flight mass spectrometry

## Abstract

*Mycobacterium abscessus* is a ubiquitous rapid-growing mycobacterium that belongs to a diverse group of non-tuberculous mycobacteria. It can cause frequent and serious infections in patients with or without risk factors. This article reports a case of disseminated *M. abscessus* in an immunocompetent Omani male with native joint septic arthritis.

## Highlights

• This case report describes the first case of disseminated *Mycobacterium abscessus* in an immunocompetent adult, and the second reported case of *M. abscessus* causing native joint septic arthritis.
• Disseminated *M. abscessus* treatment was given.

## Introduction

*Mycobacterium abscessus* is a ubiquitous rapid-growing mycobacterium (RGM) that belongs to a diverse group of non-tuberculous mycobacteria (El Helou et al., 2013). It is an environmental organism that can cause frequent and serious infections in patients with or without risk factors (Chetchotisakd et al., 2000).

The following case report describes a case of disseminated *M. abscessus* in an immunocompetent Omani male with native joint septic arthritis.

## Case report

A 63-year-old Omani male presented to the Emergency Department at Armed Forces Hospital, Muscat, Oman in 2020 with a 1-month history of persistent right knee pain, swelling, inability to bear weight and limited range of motion (ROM) associated with intermittent fever. He had attended another clinic multiple times in the past with right knee symptoms. He had been diagnosed with advanced osteoarthritis of the right knee, and treated on multiple occasions over a 2-month period with intra-articular injections of standard linear hyaluronic acid. He had also received an injection of hydrocortisone recently. A few days after hydrocortisone administration, his symptoms started to worsen. The pain became severe and he was no longer able to bear weight on his right leg. He was seen in the Emergency Department on two occasions prior to admission for assessment of worsening symptoms. He had underlying type 2 diabetes (controlled by oral antihyperglycaemic agents), hypertension and dyslipidaemia.

On admission, physical examination revealed mild redness with varus deformity of the right knee and fixed flexion deformity of 20^o^. There was significant swelling, effusion, restricted ROM due to pain, warmth, tenderness on direct pressure, and crepitus. Investigations revealed neutrophilic leukocytosis and raised C-reactive protein (CRP). Initial radiographs showed advanced osteoarthritis with calcification of the medial meniscus. Ultrasound indicated synovial thickening with septations, consistent with a diagnosis of septic arthritis. Right knee aspiration performed under aseptic technique yielded 50 mL of turbid yellowish fluid with a white cell count of 64,000 per mL. Gram staining showed Gram-positive bacilli, and Ziehl–Neelsen staining showed acid-fast bacilli. However, molecular tests (GeneXpert) did not detect *Mycobacterium tuberculosis* DNA, indicating that the organism was likely to be a non-tuberculous mycobacterium (NTM). The same organism was recovered from a blood culture submitted on admission.

The patient underwent right knee arthroscopic debridement and lavage. Additional samples were submitted for histopathological and microbiological analysis. Intravenous co-amoxiclav was commenced. His symptoms improved significantly in terms of pain, swelling and ROM; however, 7 days after initial surgery, the patient experienced worsening pain, declining ROM, and CRP began to rise again. A second arthroscopic debridement and lavage was performed.

The NTM was identified as *M. abscessus* using matrix-assisted laser desorption/ionization-time of flight mass spectrometry (MALDI-TOF MS) within the first week of admission. Antimicrobial treatment was adjusted based on culture results, and the patient received intravenous amikacin and tigecycline, and oral linezolid and azithromycin. The planned duration of treatment was 2 months. This regime led to clinical improvement, CRP declined and subsequent blood cultures yielded no growth of *M. abscessus*.

The patient left the hospital against medical advice after receiving these antibiotics for 2 weeks. He was discharged on linezolid and clarithromycin with follow-up with the infectious diseases (ID) team.

The patient was re-admitted under the ID team 1 week after leaving the hospital in order to continue his treatment, and he was restarted on the previous in-hospital regimen (intravenous amikacin and tigecycline, and oral linezolid and azithromycin). During hospitalization, he developed pancytopenia so linezolid was stopped. The patient continued taking these antibiotics for 3 weeks, and was discharged on request due to family commitments. This time, he was started on clofazimine and advised to continue oral clofazimine and azithromycin as an outpatient.

## Discussion

This case report describes a patient with disseminated *M. abscessus* infection which was isolated from both blood and synovial fluid. A positive blood culture with *M. abscessus* is one of the criteria defining *M. abscessus* disseminated infection (Fukui et al., 2015).

Although *M. abscessus* can cause localized infection regardless of immune status, disseminated infection is very rare in immunocompetent patients (Chetchotisakd et al., 2007). Immunosuppression has been suggested previously as an important risk factor for disseminated *M. abscessus* infection (Fukui et al., 2015; Johansen et al., 2020). However, the current case was not immunocompromised apart from other comorbidities. It is worth mentioning that he was not tested for anti-interferon-γ autoantibody.

Evidence for the source of infection was not identified, but it could be related to the intra-articular injections that the patient received prior to presentation. Intra-articular joint injection seems to play a crucial role in septic arthritis caused by *M. abscessus,* especially in prosthetic joints (Wang et al., 2011; Fukui et al., 2015). The presence of a prosthesis may contribute to the pathophysiology and attachment of this organism (Fukui et al., 2015). Contamination of the medication during mixing and preparation, and during the injection procedure has been reported. A greater number of intra-articular injections has also been associated with higher risk of NTM infection (Jung et al., 2015). The case patient received multiple intra-articular injections before presentation, but – unlike previous reported cases – he did not have a prosthesis.

A review of the literature yielded a single case of disseminated *M. abscessus* infection with native joint septic arthritis. Unlike the current case patient who was immunocompetent, this patient was on corticosteroids for 17 years for anti-synthetase-syndrome-associated interstitial lung disease. After 6 weeks of clarithromycin, amikacin and imipenem/cilastatin, the patient died (Fukui et al., 2015). *M. abscessus* has only been reported as the cause of osteomyelitis in two cases (Garcia et al., 2013).

Isolation of *M. abscessus* can be a challenge, especially in cases where this organism is not suspected. RGM should be identified to species level, and antibiotic susceptibility testing is highly recommended for all *M. abscessus* because of variable in-vitro susceptibilities (Griffith et al., 2007). The organism was further identified via MALDI-TOF MS, 16sRNA and InnoLippa. Unfortunately, due to the coronavirus disease 2019 pandemic, liaison with an outsourced reference laboratory outside Oman for antibiotic susceptibility testing was extremely difficult, and this is one of the challenges associated with this organism.

Treatment of *M. abscessus* can be extremely difficult and challenging as it is intrinsically resistant to many antibiotics that can be used to treat other NTM (Nessar et al., 2012). *M. abscessus* is considered to be one of the most virulent and chemotherapy-resistant RGM (Petrini, 2006).

Musculoskeletal infection caused by *M. abscessus* is usually treated with a combination of oral and parenteral antibiotics. Prolonged duration of antibiotic therapy should be warranted in *M. abscessus* infections to prevent disease progression and relapse (Griffith et al., 2007). The present case patient underwent both medical and surgical treatment.

Disseminated *M. abscessus* infection is associated with a high mortality rate (up to 48%) (Fukui et al., 2015). In contrast to the only reported case of disseminated *M. abscessus* infection with native joint septic arthritis (Fukui et al., 2015), the present case patient is still alive. This can be explained by patient immune status, early detection of *M. abscessus*, and early initiation of an appropriate combination of antibiotics.

## Conclusion

Disseminated *M. abscessus* infection is associated with a high mortality rate. Early detection and initiation of appropriate antibiotics are crucial to avoid undesirable consequences. The increasing number of NTM infections should raise suspicion of possible sources of infection, especially in patients with risk factors.

## Conflict of interest statement

None declared.
